# Recognizing Catatonia in Medically Hospitalized Older Adults: Why It Matters

**DOI:** 10.3390/geriatrics3030037

**Published:** 2018-06-29

**Authors:** Jordi Serra-Mestres, Walter Jaimes-Albornoz

**Affiliations:** 1 Department of Old Age Psychiatry, Central and North West London NHS Foundation Trust, Woodland Centre, Hillingdon Hospital, Uxbridge, Middlesex UB8 3NN, UK; 2 Psychiatry Service, Hospital Universitario Donostia, Osakidetza-Basque Health Service, E-20014 San Sebastian, Spain; walter.jaimesalbornoz@osakidetza.eus

**Keywords:** catatonia, older adults, general hospital

## Abstract

Catatonia is a neuropsychiatric syndrome characterized by a variety of motor, behavioral, emotional, and autonomic abnormalities caused by general medical, neurological, and psychiatric disorders, as well as by medications and drugs of abuse. Although there has been a plethora of research on catatonia over the last twenty years, it is still underdiagnosed. Studies of catatonia involving older adults have been sparse, despite its apparent high prevalence, higher risk of serious complications, and of association with non-psychiatric causes. This paper aims to provide an introduction to catatonia as a syndrome, as well as an account of its specificities in older adults, especially those in general hospitals, with the aim to raise awareness of catatonia amongst clinicians working with this age group in acute medical settings, so improvements in its diagnostic rates, treatment, and outcomes can be achieved.

## 1. Introduction

Catatonia is characterized by a variety of motor, behavioral, emotional, and autonomic abnormalities caused by general medical, neurological, and psychiatric disorders, and by medications and drugs of abuse [1]. The contemporary concept was introduced by the neuropsychiatrist Karl Ludwig Kahlbaum in 1874 [2]. He described stupor, mutism, rigidity, negativism, catalepsy, and echophenomena in a group of patients with various psychiatric and physical disorders. Kraepelin [3] incorporated catatonia into his concept of dementia praecox (later called schizophrenia) and catatonia was considered a part of schizophrenia. In the late 20th century, a renewed interest in catatonia began [1] situating it again in its rightful place amongst the major neuropsychiatric syndromes.

Notwithstanding the increasing amount of research on catatonia, there have been few studies involving older adults despite its high prevalence, risk of serious complications, and association with non-psychiatric causes in this age group [4,5]. Hence, it is essential that catatonia in older adults in medical settings is detected to improve diagnostic rates, treatment, and outcomes. This paper will consider the specificities of catatonia in older adults, especially in general hospitals, to raise awareness amongst clinicians working with this age group, so better outcomes can be achieved and complications avoided.

## 2. Method

A literature search in MEDLINE/PubMed was undertaken from its first registers to April 2018 for suitable articles using the search strategy “Catatonia” and “older adults”, and “general hospital”, and “geriatric”. Additionally, a manual revision of the literature referred to in the identified articles was undertaken in order to obtain old or non-indexed references. All articles were considered for inclusion if their content was felt to be relevant to this review.

## 3. General Aspects of Catatonia

Catatonia tends to present acutely and often be seen in emergency departments and in medically hospitalized patients who suddenly deteriorate and stop talking and engaging, becoming stuporous, not eating and drinking. However, in some cases, it may adopt a sub-acute onset and a chronic course. Around forty signs of catatonia have been described [6], but its cardinal ones are mutism, catalepsy/posturing, stupor, rigidity, waxy flexibility, stereotypies/mannerisms, and echophenomena [1].

Two clinical varieties of catatonia have been consistently found [1]. A hypokinetic variant or retarded-stuporous, characterized by reduced movement (it could cause full immobility), mutism, and withdrawal, most frequently found in depressive disorders and general medical conditions. A hyperkinetic or excited variant, characterized by increased aimless motor activity (qualitatively different from the over-activity of pure mania, which is purposeful), confusion, and frequent autonomic dysfunction, which is mostly seen during manic episodes and in a special form of catatonia called delirious mania. Both forms of catatonia can co-exist in the same patient, occurring in quick succession. 

Factor analysis studies of catatonic signs suggest that the dichotomy hypokinetic/hyperkinetic may not be sufficient to characterize the full syndrome. Thus, four dimensions have been described [7,8,9]:

- Catatonic excitement (mainly observed in pure mania and mixed mania)
- Abnormal involuntary movements (mainly observed in schizophrenia with stereotypies, echophenomena, posturing/catalepsy, negativism, mannerisms, Mitgehen, and Mitmachen, and also in Tourette’s disorder, and perhaps obsessive-compulsive disorder
- Volitional disturbance/catalepsy (mainly observed in schizophrenia and mixed mania)
- Catatonic inhibition (mainly observed in depression and medical catatonia)

The clinical presentation of catatonia in older adults, despite the significantly different rates of the various etiologies, is similar to that of patients in other age groups [5,10,11].

## 4. Etiology

Having diagnosed catatonia, an exhaustive search for its cause should start. These etiologies range from primary psychiatric disorders to general medical and neurological disorders, and drugs and substances of abuse [1]. Amongst the psychiatric causes, affective disorders are the most common (bipolar affective disorder and depression) [1,3,12]. Amongst the medical ones, the list is very extensive. Table 1 provides a non-exhaustive list of etiologies. From studies exploring catatonia in older adults, the most frequent diagnoses associated with catatonia were, in no special order, stroke, encephalitis, hyponatremia, urinary tract infection, depression, chronic psychosis, and dementia [5,10,11,13,14]. In three series, all cases presented with at least one vascular risk factor, and in over 50% of cases, there was recent or current exposure to antipsychotic drugs [5,10,14]. In the case of dementia, the available literature would suggest that it tends to present with another disorder, medical or psychiatric, when associated with catatonia [5,10,11,14]. An increasingly recognized cause of catatonia is an encephalitis as a result of antibodies against the NMDA receptors; an autoimmune disorder in which catatonia is found very frequently, often accompanied by significant autonomic dysfunction [15], and that it can also occur in older patients.

## 5. How Prevalent Is Catatonia?

Its prevalence is largely dependent on being recognized and the setting, but catatonia is, despite its dramatic presentation and risk of complications (sometimes life-threatening), still underdiagnosed and undertreated [1,16]. 

The frequency of catatonia attributed to general medical conditions is 21–46% of all catatonia cases [13,17,18]. Catatonia has also been reported to cause diagnostic dilemmas in unresponsive patients attending emergency departments [19], where its frequency due to a general medical condition is up to 41% of all cases [20]. Catatonia has also been reported in intensive care with a prevalence of 3.8% [21], and is reported in a significant proportion of cases of encephalitis due to anti-NMDAr antibodies [15].

In adults over the age of 65, a review of 71 cases found that 28.16% of cases of catatonia were associated with a general medical condition [13]. In two studies of older adults in general hospitals, catatonia was found in 5.5–8.9% of cases [5,10]. In acute psychogeriatric settings, two studies have reported rates of catatonia in patients with dementia of 4.7–12.6% (using the Diagnostic and Statistic Manual-5 (DSM-5) [22] and the Bush-Francis Catatonia Rating Scale (BFCRS), respectively) [11], and 42.8% [14] (using Fink and Taylor [1] and DSM-5 criteria [22]). In older patients with psychiatric disorders in acute psychiatric hospitals, two studies have reported rates of catatonia of 20.8–39.6% [23], and of 6.1–11.2% [11], depending on the diagnostic criteria used. In the former study, cognitive impairment and dementia were found to have a rate of catatonia of 35.3–58.8% [23].

It has been proposed that in catatonic patients, an extensive workup to exclude a general medical cause is necessary before attributing it to a psychiatric disorder [11,19], even when there is a past psychiatric history.

The mean number of catatonic signs in medically hospitalized older patients with catatonia in two studies was of 8.8 (5–12) [5] and 5 (3–8) [10], respectively. The most frequent signs were immobility/stupor 83–100%, posturing 67–70%, mutism 33–80%, staring 50–100%, rigidity 67–90%, and withdrawal 50–80% [5,10]. In psychiatrically hospitalized older adults, the retarded-stuporous variant predominated in over 50% of cases in one study [11], and was the most frequent presentation of catatonia in those with depression (89%) [23].

## 6. Pathophysiology

The common pathophysiology of catatonia entails a dysregulation of specific neural pathways affecting motor function and emotional regulation that relate to circuitry linking the medial frontal and inferior orbital cortices to the basal ganglia and thalami, with connections to the parietal lobes, cerebellum, and limbic system [1,24,25]. Thus, catatonia would be mediated by hypofunction of cortical (frontal) Gamma-aminobutyric Acid-A (GABA-A) neurotransmission, leading to hypoactivity of subcortical dopaminergic pathways in the basal ganglia, causing immobility and stupor. This is the ‘top-down’ model of catatonia [24] and explains its retarded-stuporous variant. The neuroleptic malignant syndrome, a drug-induced form of lethal catatonia, would be mediated through an initial subcortical hypofunction of dopaminergic pathways (caused by dopaminergic blockade by antipsychotic drugs), which would then cause cortical GABA-A hypofunction (a ‘bottom-up’ mechanism) [24]. A similar dysregulation in fronto-hypothalamic circuits explains the autonomic signs observed in catatonia [25]. In addition, glutamate has also been implicated in the pathophysiology of catatonia [26], being a biological antagonist of GABA, and because glutamate antagonists improve catatonia.

Catatonia has also been conceptualized as an evolutionary response to intense fear, including that induced by psychopathological experiences such as hallucinations and delusions; a fear that causes the subject to ‘freeze’ in order to self-protect from predators stimulated by movement [27]. This freezing would correspond to the immobility, stupor, catalepsy, and mutism observed in catatonia. Alternatively, a catatonic excitement would be analogous to the ‘fight–flight’ response mediated by the sympathetic nervous system.

## 7. Assessment and Diagnosis

Assessment is mostly undertaken by careful observation during the clinical interview and by eliciting specific signs during the neurological examination. Although there is no consensus on how long the signs must be present for a diagnosis, at least one hour during a day has been suggested, or when signs can be reproduced on two or more occasions [1]. The DMS-5 states that a diagnosis of catatonia can be made if there are at least 3 core signs out of 12 present at the time of assessment [22].

In Table 2, the most frequent signs of Catatonia are enumerated, as well as their definition and mode of assessment. This is based on the Bush–Francis Catatonia Rating Scale [28].

Fink and Taylor [1] have also proposed a set of criteria in which timings for duration of signs, as well as a “hierarchy” of signs, is given, reflecting that some signs are more important than others for diagnostic purposes (at least two signs to be present for a diagnosis): (A) immobility, mutism, or stupor for a duration of at least one hour, associated with at least one of the following; catalepsy, automatic obedience, or posturing, observed or elicited on two or more occasions. (B) In the absence of immobility, mutism, or stupor, at least two of the following, which can be observed or elicited on two or more occasions; stereotypy, echophenomena, catalepsy, automatic obedience, posturing, negativism, or ambitendency.

The routine use of validated rating scales has also been advocated to facilitate identification of catatonic signs and a diagnosis of catatonia [1,25]. The Bush–Francis Catatonia Rating Scale [28], which consists of a screening instrument (14 items that are scored as present or absent) and a full severity scale (14 plus 9 other items scored 0–3) has been widely recommended for its ease of use and because of its reliability and validity [29]. In this scale, the presence of two or more signs is suggestive of catatonia [28].

A specific screening method for identifying catatonia in acute medical settings has recently been proposed with the mnemonic “A SLIME-posture” [30]:

- Acute or subacute onset
- Speech: disordered—poverty, whispering, mutism
- Latency: increased response latency in speech, affect, or movement
- Interaction (stupor): decreased and out of proportion to relatively preserved alertness
- Muscle: Increased tension/tone on examination
- Eyes: staring
- Posture: posturing, including grimacing

## 8. Differential Diagnosis

In a general hospital or emergency room setting, a considerable number of conditions that cause stupor and/or abnormal movements will need to be considered. Catatonia should be always thought of in the differential diagnosis of altered states of awareness in emergency departments and acute medical settings [19]. Diagnoses that will need excluding are non-convulsive status epilepticus, delirium, cerebral trauma, acute cerebrovascular disease, locked-in syndrome, stiff-person syndrome, “off” periods in Parkinson’s disease and its akinetic-rigid form, malignant hyperthermia, selective mutism, factitious disorder, maladaptive coping with medical illness with non-cooperation and contrary behavior, and malingering [1,30,31]. In epilepsy, catatonia has been reported in ictal, post-ictal, and interictal states, and also as a result of anticonvulsant treatment and of alternative psychoses [32].

## 9. Special Forms of Catatonia

### 9.1. Malignant/Lethal Catatonia

Characterized by the sudden development of intense excitement, hyperthermia, catalepsy/posturing, mutism, rigidity, and stereotypies, in the context of delirium; it represents the most severe form of catatonia. There is severe autonomic dysfunction with fever, tachycardia, tachypnea, and hypertension, and multiorgan dysfunction. It is associated with high mortality rates, mainly due to diagnostic delays. The treatment of choice is with benzodiazepines and electroconvulsive therapy (ECT), or both, and should always be accompanied by vital support measures and treatment of the cause of the symptoms.

### 9.2. Neuroleptic Malignant Syndrome (NMS)

NMS has been considered by many as a form of (malignant) catatonia precipitated by exposure to antipsychotic drugs [1,25]. It has been reported to occur in 0.5–1% of patients receiving antipsychotic treatment, usually within two weeks of exposure to either typical or atypical antipsychotics. A very similar syndrome has been observed with exposure to other types of drugs (e.g., toxic serotonin syndrome). Treatment is with lorazepam and ECT [1,25]. Clinical manifestations include rigidity leading to muscle breakdown and renal failure, hyperthermia, altered consciousness, autonomic dysfunction, mutism, staring, negativism, posturing, verbigeration, and echophenomena.

### 9.3. Periodic Catatonia

Recurrent periods of hypokinetic catatonic signs alternating with periods of hyperkinesis, lasting between 4–10 days, and recurring over a period of months or years with symptom-free intervals. Some patients may display grimacing, stereotypies, and negativism in the inter-ictal intervals, especially as they grow older. It is rare and has been observed to segregate within families in an autosomal dominant pattern. There is a linkage to chromosome 15q15 [33]. One case has been described of a 22.q13.3 chromosomal microdeletion [18]. 

### 9.4. Delirious Mania

Characterized by an acute onset of excitement, grandiosity, emotional lability, delusions, insomnia, and other symptoms typical of mania, and accompanied by delirium. There is often fever and other autonomic dysfunctions. It can be made worse by the incorrect diagnosis of an acute psychosis or of delirium with the subsequent administration of antipsychotics [34].

## 10. Catatonia and Delirium

Delirium in patients with catatonia has been increasingly described [5,10,14,23,35,36]. Both syndromes share clinical features, lack specific laboratory findings and biomarkers, and are diagnosed on clinical grounds [35]. Delirium is a prominent clinical manifestation of malignant catatonia, delirious mania, and NMS [30]. In two studies, 30–50% of cases of older adults in acute medical settings with catatonia were found to suffer from co-existing delirium [5,10], whilst in another study of 205 patients with delirium in a general hospital (19% of the sample was older than 65 years of age), catatonia was present in 12.7–30.2% of cases, depending on the diagnostic criteria used [36]. In the first two studies [5,6,7,8,9,10], the clinical presentation of catatonia was generally in the retarded-stuporous form. In the third study [36], mixed and hypoactive delirium subtypes were more frequently encountered in patients with catatonia. Patients with catatonia were also found to have a higher frequency of perceptual disturbances, motor retardation, and onset of delirium prior to admission to hospital [36]. In another study in acute psychogeriatric inpatients, 66.7% of cases who on admission had two or more catatonic signs in the BFCRS, also had delirium [23].

The differential diagnosis between catatonia and delirium is challenging as both present with prominent psychomotor abnormalities. Delirium’s classification predominantly relates to its motor aspects, thus divided in hyperactive, hypoactive, and mixed forms [36]. There are also hypoactive and hyperactive forms of catatonia. This can be misdiagnosed as delirium and managed as such, and whilst delirium appears in the list of differential diagnoses of catatonia, the latter rarely figures in that of delirium [35].

The causes of both delirium and medical catatonia greatly overlap [36]. There are, however, differences in their pharmacological management. The treatment of choice in catatonia, lorazepam, is rarely the treatment of choice in delirium except when caused by benzodiazepine withdrawal. The most widely used symptomatic treatment of choice in delirium, antipsychotics, is generally to be avoided in the management of catatonia. Therefore, the ability of clinicians to ascertain a catatonic dimension in cases of delirium will facilitate the selection of the right treatment [36]. 

On a retrospective chart review of adults with catatonia in a general hospital, 40% had a psychiatric history, 50% had suspected delirium, and 60% had dopamine antagonist exposure prior to catatonia onset [17]. In these patients, an older age, the presence of suspected delirium, and first episode of catatonia, were associated with a greater number of diagnostic tests during admission [17]. Patients with a psychiatric history were less likely to have suspected delirium, and they received less diagnostic tests [17]. 

## 11. Investigations

Since medical causes of catatonia will be very significant in older patients in a general hospital, a psychiatric cause should not be attributed initially, and adequate investigations should be undertaken. In addition to a comprehensive physical and a neurological examination, appropriate hematological and biochemical tests will be required, ranging from full blood count and inflammatory markers; to kidney, liver, and thyroid function tests; calcium; glucose; proteins; and creatine-kinase. A blood and/or urine toxic screen may be needed, as well as a microbiological urine examination [1,25,37]. Depending on the presentation, vasculitic or encephalitic processes may be suspected, and other plasma and/or cerebrospinal fluid (CSF) tests may be required, including various serologies and autoantibody screens. Structural brain imaging will often be required to exclude central neurological pathologies. An electroencephalogram (EEG) may be needed to exclude a non-convulsive status epilepticus presenting with ictal catatonia. 

No specific biochemical or hematological abnormalities have been described in catatonia. In malignant catatonia and in NMS, raised leucocytes and creatine-kinase, and low serum iron have often been described [25]. A raised creatine-kinase should alert to severe rigidity and muscle breakdown that can lead to serious complications. A workup for medical catatonia is fully described in a recent evidence-based medicine monograph on catatonia in medically ill patients [30].

## 12. Complications

Prolonged catatonia is associated with adverse events that can be life-threatening. Complications tend to be caused by the effects of prolonged immobility, withdrawal causing poor or no food and fluid intake, or autonomic dysfunction. The more severe and longer lasting the catatonia, the higher the risk of complications. Older patients with undiagnosed catatonia have been reported to be at an increased risk of major complications and adverse events [4]. In a retrospective review [38], the frequency of significant complications was of 11%. In the same series, the rate of deep venous thrombosis was 6% and of pulmonary embolism was 2%. In a prospective study of older patients with catatonia in a general hospital, the rate of complications observed was 40% and the mortality rate was 20% [5]. 

Catatonic patients should be managed in hospitals with specialist multidisciplinary input to ensure treatment of the cause of catatonia; good hydration and nourishment; and prevention of infections, pressure ulcers, contractures, and thrombosis. Table 3 lists the most frequent complications associated with catatonia.

## 13. Medical Management

Catatonia is a treatable condition. The therapeutic approach is three-fold. First, the symptomatic treatment of the catatonia itself; second, the treatment of its cause; and third, the supportive measures to prevent complications. Clinicians must be mindful that patients in a state of catatonia are often unable to consent to treatment and that a legal framework to provide this in their best interests should be in place.

Despite the paucity of randomized controlled trials (RCTs), the available literature overwhelmingly suggests that benzodiazepines (in particular lorazepam, but also diazepam, clonazepam, and oxazepam) are very effective in improving catatonia, and they are safe in most patients [30]. This is believed to be through their affinity and effect on GABA-A receptors [37]. Treatment can be dramatically effective, with a complete resolution of signs in 60–80% of acute cases [39]. Treatment should then continue until the treatment for the cause of catatonia is well under way and there are no further signs of catatonia.

The principles of treatment of catatonia are outlined in Box 1, and are adapted to an older adult population [30,37].

Other pharmacological interventions are also available if lorazepam or other benzodiazepines cannot be administered, or as augmentation strategies. The evidence base is currently very limited. These other drugs are also normally administered orally and may not be ideal for those requiring other routes of administration, and tend to be slower in the onset of their effect with the exception of zolpidem (another GABA-A agonist). The following drugs can provide beneficial effects: amantadine and memantine (NMDA receptor antagonists, although it is possible that the effect is via enhancement of dopamine levels in frontal cortex and striatum [30]), bromocriptine, sodium valproate, carbamazepine, and topiramate (see [30] for review).

ECT is also very effective. Eighty to one hundred percent of patients with catatonia respond to ECT [40]. In the United Kingdom, it is approved by the National Institute for Health and Care Excellence (NICE) for the treatment of catatonia [41]. ECT should be considered as first line in malignant catatonia, NMS, and delirious mania [40]. It should also be considered in catatonia unresponsive or partially responsive to benzodiazepines [40].

Box 1 **Principles of Pharmacological Management of Catatonia.**

- Always consider the diagnosis of catatonia
- Review the medication regime (especially for dopamine antagonists such as antipsychotic drugs or metoclopramide) and carry out a physical examination and investigations to identify the cause
- Start treatment for the cause of catatonia as soon as possible
- Administer a therapeutic challenge of lorazepam 0.5–1 mg po/IM. If ineffective, repeat again after 30 min, and then again in another 3 h
- An initial positive response usually predicts a more sustained response with further doses and would confirm the diagnosis in 80% of cases
- The dosage of lorazepam that was effective in resolving the symptoms should be continued until treatment of the primary disorder is well underway. In older persons, usually 0.5 mg three to four times a day
- A premature suspension of treatment with lorazepam is likely to result in a recurrence of catatonic signs
- Dose titration should be against sedation
- Lorazepam should be switched to oral administration as soon as possible if intramuscular or intravenous administration had been started first
- A course of electroconvulsive therapy (ECT) should be considered if there is minimal or no response to lorazepam
- If the primary cause of catatonia is a psychotic illness, antipsychotics may be re-introduced when the signs of catatonia have been controlled

## 14. Prognosis

Catatonia tends to have a good prognosis if it is detected early, its cause is treated, its symptoms managed, and complications avoided. In medical catatonia, metabolic causes may have a better prognosis than structural brain lesions [31]. Older adults in general hospitals with a longer duration of untreated catatonia may have a worse outcome in terms of complications [5].

## 15. Conclusions

Catatonia is not infrequent in older adults in acute general medical settings, where its non-psychiatric causes can represent up to almost a third of all cases [13], and where it can be associated with delirium in up to 50% of cases [5,10]. Undiagnosed and untreated catatonia can lead to an increased risk of complications and mortality [4,5], and it is therefore paramount that clinicians working with older adults in these settings have an increased awareness of this condition in order to implement the appropriate treatment for the symptoms of catatonia and its causes.

## Figures and Tables

**Table 1:** Causes of Catatonia (not exhaustive).

Psychiatric and neurodevelopmental	Mania and depression (Bipolar disorder), unipolar depression, late-onset depression, Schizophrenia, and chronic psychoses Anxiety disorder, dissociative disorder and Ganser syndrome, adjustment disorders, acute stress reactions, obsessive-compulsive disorder, Prader–Willi syndrome, autistic spectrum disorders, and Gilles de la Tourette syndrome
Neurological	Cerebrovascular disease Bilateral infarction of the parietal lobes, temporal infarcts, thalamic lesions, bilateral lesions in globus pallidus Anterior cerebral and anterior communicating artery aneurysms and hemorrhagic infarcts, subdural hematoma Hydrocephalus Frontal lobe traumatic contusions and neoplasms, paraneoplastic encephalopathy, and malignant and benign central nervous system tumors Encephalitis (including anti-NMDAr, herpes, Human Immunodeficiency Virus (HIV), post-immunisation, and Encephalitis Lethargica), meningitis, and cerebral abscesses Post-encephalitic states, especially with Parkinsonism, Progressive Multifocal Encephalopathy Neurosyphillis, other central nervous systeminfections: typhoid fever, tuberculosis, borreliosis, malaria, trypanosomiasis, hidatidosis Parkinson’s disease and Lewy Body disease Frontotemporal dementia, Alzheimer’s disease, vascular dementia, Creutzfelt-Jakob disease, Fatal Familial Insomnia Motor Neuron Disease, Wilson’s disease, Huntington’s disease, multiple sclerosis, Progressive Supranuclear Palsy Epilepsy (absence seizures, complex non-convulsive partial seizures, generalised and complex partial (focal) status epilepticus, post-ictal states) Brain trauma acute and sequelae, Wernicke’s encephalopathy, hepatic encephalopathy, central pontine myelinolisis Narcolepsy, Tay-Sachs disease, Tuberous sclerosis
Metabolic and endocrine, haematological and immune	Diabetic ketoacidosis, hypercalcemia, renal failure, liver failure Acute intermittent porphyria, homocystinuria, membranous glomerulonephritis, hyponatremia, hypernatremia Lysosomal disease, hypothyroidism, hyperthyroidism, hyperparathyroidism, hypoglycemia, Sheehan’s syndrome Addison’s disease, Cushing’s disease, syndrome of inappropriate antidiuretic hormone secretion (SIADH) Vitamin B12 deficiency, nicotinic acid deficiency, pellagra Systemic Lupus Erythematosus, Pediatric Autoimmune Neuropsychiatric Disorder Associated to Streptococcal Infection (PANDAS), antiphospholipid syndrome, renal and hepatic transplant, Langerhans carcinoma
Pharmacological, toxic and other	Typical and atypical antipsychotics (use and withdrawal) including clozapine, levodopa, amantadine, serotonergic drugs (selective serotonin reuptake inhibitors (SSRIs), trazodone, venlafaxine, etc.), lithium, acetyl-cholinesterase inhibitors Cephalosporines, ciprofloxacin, levofloxacin, azitromicine, levetiracetam, sodium valproate, gabapentin Disulfiram, paracetamol, aspirin, tramadol, hydroxicine, antiretroviral, ACTH, steroids Cyclosporine, chlorphenamine, methylphenidate, morphine, methadone, meperidene, allopurinol Benzodiazepine withdrawal, cocaine, cannabis, LSD, mescaline, ketamine, phenylcyclidine, amphetamines, organophosphates, ethylene, carbon monoxide, severe burns

**Table 2:** Catatonic semiology and its evaluation.

Sign	Definition	Mode of Evaluation
Excitement	Extreme and constant hyperactivity without aim. May be destructive	Observation
Immobility and stupor	Extreme hypoactivity, complete immobility, minimal response to stimuli	Observation Stimulation
Mutism	Minimal or absent verbal response	Observation Conversation
Fixation of gaze	Fixed gaze with little or absent response to environment and reduced blinking	Observation
Posturing Catalepsy	Maintenance of a mundane or bizarre posture, for long periods of time, even when uncomfortable Includes the *psychological pillow*, where a patient lies in a supine position with their head raised as if resting on a pillow Same as above but patient is positioned by the examiner	Observation Placing arm, leg, or body of patient in a particular position
Grimacing	Production and maintenance of bizarre facial expressions Includes *Schnauzkrampf*, where the lips are drawn up and out in a puckered position	Observation
Echopraxia and echolalia	Imitation of the movements or speech of a third party or of the examiner	Observation Conversation Examiner scratches own head in an exaggerated manner
Stereotypies	Aimless and repetitive motor activity (the abnormality is not inherent to the act but to its frequency) *Verbigeration* is a vocal stereotypy where the patient will repeat a phrase or sentence constantly	Observation
Mannerisms	Directed motor activity undertaken in a strange or exaggerated form (e.g., jumping, tiptoeing, waving at passersby, etc.)	Observation
Verbigeration	Production and repetition of sentences or words	Observation Conversation
Rigidity	Maintenance of a rigid posture despite attempts to being moved (exclude if tremor or cogwheeling exist)	Examination of muscle tone
Negativism	Unmotivated resistance to instructions to or attempts to move or examine the patient, or conduct opposite to that required	Examination of muscle tone Verbal instructions
Waxy flexibility (*flexibilitas cerea*) or waxy rigidity	Initial resistance to passive movement of a limb followed by a facilitation of the movement (similar to the feeling of folding a hot candle)	Examination of muscle tone
Withdrawal	Refusal to eat, drink, or to sustain others’ gaze	Observation Feeding
Impulsivity	The patient suddenly behaves inappropriately without reason (undressing, running down the corridor, shouting). After the event, unable to explain their behavior	Observation
Automatic obedience	Exaggerated cooperation with the examiner’s requests, or repetition of movements or actions that were only required once	Examiner puts hand in pocket whilst saying to patient: “Stick out your tongue; I am going to prick it with a pin”
Passive obedience (Mitgehen)	Raising of the arm in response to a slight pressure by the examiner’s finger despite the instruction not to raise it	Ask patient to extend their arm, place finger under patient’s palm and try to raise their arm by gentle pressure whilst giving the instruction: “Don’t raise your arm”
Gegenhalten	Resistance to passive movement that is proportional to the strength of the stimulus.	Passive mobilization of a limb
Ambitendency	The patient seems to get stuck in indecisive conduct or movements	Observation Give hand to patient as intending to shake their hand whilst instructing: “Don’t shake my hand”
Grasping	Grasping reflex that occurs when the patient’s palm is stimulated	Examination of the grasping reflex
Perseveration	Repetitive returning to the same topic or movement	Observation Conversation Giving instructions
Combativeness	Unmotivated, undirected, and unexplained combative behavior	Observation
Autonomic abnormalities	In temperature, blood pressure, pulse, respiratory frequency. Also, inappropriate perspiration. Incontinence (urine/fecal)	Observation Checking of vital signs Checking of clinical observations charts

**Table 3:** Common complications in Catatonia [31,38].

**Vascular**	Thrombophlebitis, deep venous thrombosis, disseminated intravascular coagulation
**Cardiac and respiratory**	Myocardial infarction, cardiac or respiratory arrest, aspiration, pneumonia, pneumonitis, pulmonary thromboembolism
**Renal and urinary**	Renal failure, urinary retention, urinary incontinence, bacteriuria, urinary tract infection
**Gastrointestinal, endocrine and electrolytic**	Haemorrhage, dehydration, hypernatremia, hyponatremia, malnutrition, chachexia, liver damage, hypoglycaemia
**Neurological and muscular**	Muscle contractures, rhabdomyolysis, neuropathies secondary to posture, convulsions
**Other**	Sepsis, oral candidiasis, skin infections, pressure ulcers, burns
